# The association between the presence and burden of periodic discharges and outcome in septic patients: an observational prospective study

**DOI:** 10.1186/s13054-023-04475-w

**Published:** 2023-05-09

**Authors:** Lorenzo Ferlini, Christelle Maenhout, Ilaria Alice Crippa, Armin Alvaro Quispe-Cornejo, Jacques Creteur, Fabio Silvio Taccone, Nicolas Gaspard

**Affiliations:** 1 Department of Neurology, Erasme Hospital, Université Libre de Bruxelles, Route de Lennik, 808, 1070 Brussels, Belgium; 2 Department of Anesthesiology and Intensive Care, Policlinico San Marco, Gruppo San Donato, Zingonia, Italy; 3 Department of Intensive Care, Erasme Hospital, Université Libre de Bruxelles, Brussels, Belgium; 4 Department of Neurology, Yale University School of Medicine, New Haven, CT USA

**Keywords:** Sepsis, Brain dysfunction, Periodic discharges, Outcome and EEG

## Abstract

**Background:**

Sepsis-associated encephalopathy (SAE) is frequent in septic patients. Electroencephalography (EEG) is very sensitive to detect early epileptic abnormalities, such as seizures and periodic discharges (PDs), and to quantify their duration (the so-called burden). However, the prevalence of these EEG abnormalities in septic patients, as well as their effect on morbidity and mortality, are still unclear. The aims of this study were to assess whether the presence of electrographic abnormalities (i.e. the absence of reactivity, the presence and burden of seizures and PDs) was associated with functional outcome and mortality in septic patients and whether these abnormalities were associated with sepsis-associated encephalopathy (SAE).

**Methods:**

We prospectively included septic patients, without known chronic or acute intracranial disease or pre-existing acute encephalopathy, requiring ICU admission in a tertiary academic centre. Continuous EEG monitoring was started within 72 h after inclusion and performed for up to 7 days. A comprehensive assessment of consciousness and delirium was performed twice daily by a trained neuropsychologist. Primary endpoints were unfavourable functional outcome (UO, defined as a Glasgow Outcome Scale-Extended—GOSE—score < 5), and mortality collected at hospital discharge and secondary endpoint was the association of PDs with SAE. Mann–Whitney, Fisher’s exact and *χ*^2^ tests were used to assess differences in variables between groups, as appropriate. Multivariable logistic regression analysis with in-hospital mortality, functional outcome, SAE or PDs as the dependent variables were performed.

**Results:**

We included 92 patients. No seizures were identified. Nearly 25% of patients had PDs. The presence of PDs and PDs burden was associated with UO in univariate (*n* = 15 [41%], *p* = 0.005 and *p* = 0.008, respectively) and, for PDs presence, also in multivariate analysis after correcting for disease severity (OR 3.82, IC 95% [1.27–11.49], *p* = 0.02). The PDs burden negatively correlated with GOSE (Spearman’s coefficient *ρ* = − 0.2, *p* = 0.047). The presence of PDs was also independently associated with SAE (OR 8.98 [1.11–72.8], *p* = 0.04). Reactivity was observed in the majority of patients and was associated with outcomes (*p* = 0.044 for both functional outcome and mortality).

**Conclusion:**

Our findings suggest that PDs and PDs burden are associated with SAE and might affect outcome in septic patients.

**Supplementary Information:**

The online version contains supplementary material available at 10.1186/s13054-023-04475-w.

## Background

Sepsis is defined as a life-threatening organ dysfunction caused by a dysregulated host response to infection [1] and represents a major cause of morbidity and mortality worldwide [2]. Sepsis-associated encephalopathy (SAE) is defined as a diffuse cerebral dysfunction that accompanies sepsis in the absence of direct CNS infection, macroscopic structural abnormality or other types of encephalopathy [3]. Although it is mostly reversible, SAE is associated with a greater short-term mortality [4] and a higher risk to develop long-term cognitive impairment among survivors [5]. SAE represents the most frequent organ dysfunction associated with sepsis [6], but its occurrence varies greatly among studies, ranging from about 20 to 70% of septic patients [4, 7, 8], due to the lack of a specific diagnostic test.

Studies showed that electroencephalography (EEG) is more sensitive than clinical criteria to detect encephalopathy [9, 10], but it is not routinely employed for SAE diagnosis. Furthermore, EEG is the only available tool to detect electrographic seizures, which have been reported in 15–20% of patients with sepsis [11]. Although the lack of EEG reactivity has been associated with unfavourable outcome in different studies [12–14] in septic patients, the prevalence of other EEG abnormalities, including periodic discharges and seizures, as well as their diagnostic value to discriminate delirious from non-delirious patients and predict mortality, are still unclear. Although seizures and periodic discharges are associated with mortality in critically ill patients [9, 15], this association seems to be weaker in septic patients [11–14]. This finding may be the results of different study limitations. In particular, the prevalence, or ‘burden’, of EEG abnormalities during recording has never been considered in patients with sepsis, although it is associated with outcome in other conditions [15–17]. The notion that sepsis is a risk factor for seizures has been recently challenged in a cohort of septic patients without prior neurological history and in which no seizures occurred [14]. These results might suggest that sepsis-induced neuroinflammation and neuronal excitotoxicity due to neuronal major metabolic demand and major excitatory neurotransmitters release may trigger seizures particularly in the presence of a pre-existing cerebral injury, but this remains to be confirmed.

The primary goal of this study was therefore to assess whether cEEG abnormalities, in particular the presence and burden of seizures and PDs, were associated with functional outcome and mortality of critically ill septic patients. The secondary goal was to assess whether these abnormalities were associated with SAE.

## Methods

### Study design and population

We prospectively included all patients treated for sepsis, defined according to consensus criteria [1], in the ICU of Erasme Hospital (Bruxelles, Belgium) between January 2016 and August 2021. The local ethics committee approved the study protocol, and informed consent was obtained from the patient or her/his legal representative. This study was performed in line with the principles of the Declaration of Helsinki and in accordance with the STROBE guidelines. Inclusion criteria were age > 18 years, sepsis diagnosed from less than 48 h and expected ICU stay > 24 h. Exclusion criteria included the presence of previous chronic or acute intracranial disease, including cognitive impairment and psychiatric disorders, or pre-existing acute encephalopathy from another aetiology than sepsis (i.e., liver or renal failure), drugs or substance abuse or their complications (i.e., alcoholic cirrhosis), history of cardiac or neurosurgery or respiratory-cardiac arrest in the last 6 months, pregnancy, end-of-life care, and consent refusal to study inclusion or continuous EEG recording.

### Data collection

We collected demographic data, such as age, gender, medical history, medication, smoking, and alcohol consumption. Sepsis severity was assessed using the Acute Physiologic and Chronic Health Evaluation (APACHE) II and a modified APACHE-II (“non-neuro APACHE II “) score, without the consciousness component (Glasgow Coma Scale, GCS) [12]. Creatinine and bilirubin levels as well as the presence of an acute [18] or chronic (defined as the presence of a pre-existing estimated glomerular filtration rate less than 60 ml/min) renal or hepatic failure (defined as a bilirubin level above 1.2 md/dl [19]), white blood cells count, neuron-specific enolase (NSE) levels, natremia, ammonemia, PaCO_2_ values and body temperature were prospectively recorded on a daily basis. Use of sedation (propofol, midazolam, dexmedetomidine) and/or neuromuscular blocking agents, of vasoactive medications (norepinephrine, epinephrine, dopamine, vasopressin and dobutamine) and antibiotics (beta-lactam, cephalosporin, metronidazole vs other types), and use of mechanical ventilation or renal replacement therapies were also recorded. We defined patients in septic shock according to [1]. Hospitalization mortality was noted.

### Continuous EEG (cEEG)

cEEG was initiated as soon as possible after patient screening and was continued until ICU discharge or for up to 7 days, using 21 Ag/AgCl electrodes placed according to the international 10–20 system. An EEG reader (LF), blind to patients’ clinical condition, performed visual analysis of all the recordings for the entire population. We recorded information about background (i.e., the presence of a posterior dominant rhythm (PDR), sleep and reactivity) and about rhythmic and periodic patterns (rhythmic delta activities (RDA) and periodic discharges (PDs). Findings were reported and quantified hourly according to the 2013 version of the American Clinical Neurophysiology Society Critical Care EEG terminology [20]. Results were retrospectively corrected in accordance with the criteria of the updated 2021 version of the terminology [21]. A background was defined as suppressed if all activity had an amplitude < 10 mV, low-voltage if most or all activity < 20 mV but not qualifying as suppressed, suppression-burst as a pattern of attenuation/suppression alternating with higher voltage activity, with 50– 99% of the record consisting of attenuation or suppression, according to [21]. Reactivity was tested once daily following a standardized protocol (Additional file 1: Table S1), and EEG was classified as reactive or unreactive according to [21]. The EEG-reactivity-percentage was calculated as the ratio between the number of times an EEG was deemed as reactive and the total number of tests. Encephalopathy was graded according to a modified Synek scale (Additional file 1: Table S1). The prevalence of rhythmic and periodic patterns was calculated using the proportion of time that included those patterns in an hour. Each pattern was defined as occasional if present in less than 10% of the considered hour, frequent if present between 10 and 49%, abundant between 50 and 89% and continuous if more than 90%. The mean value of each interval (respectively, 5, 30, 70 and 95%) was used to determine the burden of the pattern. The mean burden was calculated as the hourly averaged prevalence of the pattern per patient.

### Delirium and functional assessment

Consciousness and delirium were assessed twice daily by a trained neuropsychologist with the Coma Recovery Scale-Revised (CRS-R) [22] and the Confusion Assessment Method for the ICU (CAM-ICU) [23]. Patients were scored as having SAE if their CRS-R score was lower than 23. If the Richmond Agitation-Sedation Scale [24] was lower than − 3, the CAM-ICU was considered as not assessable. Functional outcome was assessed at discharge from the hospital using the Glasgow Outcome Scale-Extended (GOSE) [25]. Poor functional outcome was defined as a GOSE < 5.

### Statistical analysis

Statistical analysis was performed using BM SPSS^®^ Statistics for Windows version 25 software (IBM, Armonk, NY, USA). *p*-value < 0.05 was considered significant. Categorical variables were expressed as a count (percentage), and continuous data were presented as median and [interquartile range (IQR)]. Mann–Whitney, Fisher’s exact and *χ*^2^ tests were used to assess differences in variables between groups, as appropriate. We also performed subgroup analyses including only non-sedated patients. Multivariable logistic regression analysis with in-hospital mortality, functional outcome, SAE or PDs as the dependent variables were performed. Only variables with a *p*-value < 0.1 in the univariate analysis were included. If variables showed co-linearity (a linear correlation coefficient > 0.3), only the one with the strongest association in univariate analysis was further considered. In order to homogenize data, since patients had different duration of EEG recordings and ICU length of stays, we considered the worst value of the CRS-R, CAM-ICU and laboratory data (including corporal temperature, white blood cells, creatinine, bilirubin, NSE levels). We calculated the cut-off of EEG-reactivity-percentage that best discriminated favourable from unfavourable outcome patients, and we considered as mostly reactive all patients presenting with an EEG-reactivity-percentage above this cut-off. The PDR was considered present if observed at least during one 1-h epoch. The Spearman’s rank-order correlation coefficient was used to measure the strength of the association between PD averaged burden or PDs frequency and GOSE.

## Results

### Study population

During the period of the study, 142 septic patients fulfilled the inclusion criteria; 50 (35%) declined the cEEG monitoring, leaving 92 patients for the final analysis. Patients’ characteristics are summarized in Table 1. Sixty-six (72%) patients developed SAE during the ICU stay. Thirty-seven patients (40%) had an unfavourable outcome at hospital discharge. Twenty-two patients (24%) died in the ICU, twenty-eight during hospitalization (30%).

**Table 1:** Clinical, laboratory and EEG variables associated with periodic discharges

	Total (*n* = 92)	Periodic discharges		*p*	Multivariate analysis		
		Absent (*n* = 69)	Present (*n* = 23)		*p*	OR	IC 95%
Age. years	65 [55.8–73.3]	66 [59–74]	62 [50–73]	0.46	–		
Female	30 (33%)	17 (25%)	13 (57%)	**0.005**	**0.006**	4.2	[1.50–11.82]
Sepsis origin							
Abdominal	37 (40%)	30 (43%)	7 (30%)	0.48	–		
Respiratory	27 (29%)	19 (28%)	8 (35%)		–		
Urinary	10 (11%)	8 (12%)	2 (9%)		–		
Soft tissue	9 (10%)	7 (10%)	2 (9%)		–		
Unknown	5 (5%)	2 (3%)	3 (13%)		–		
Other	4 (4%)	3 (4%)	1 (4%)		–		
Septic shock	42 (47%)	30 (43%)	12 (53%)	0.47			
APACHE II	22 [16–29]	21 [16–26.5]	27 [21–31]	**0.049**	**0.021**	1.07	[1.01–1.14]
Non-neuro APACHE II	18 [11–24]	18 [12–23]	19 [12–26]	0.46	–		
CRS-R	18 [2–23]	22 [6–23]	5 [0–7]	** < 0.001**	–		
Sepsis-associated encephalopathy	66 (72%)	44 (64%)	22 (96%)	**0.003**	–		
GCS	14 [6–15]	14 [10–15]	7 [3–13]	** < 0.001**	–		
CAM-ICU+	29/70 (41%)	21/58 (36%)	8/12 (67%)	0.051	–		
RASS < − 3	22 (24%)	11 (16%)	11 (48%)	**0.002**	–		
Sedation during EEG	30 (33%)	17 (25%)	13 (57%)	**0.006**	–		
Propofol dose (mg/kg/day)	20 [14.5–35.5]	15 [4.5–20]	39 [33–53]	** < 0.001**	–		
Propofol duration (days)	2 [1–4]	1 [1–2]	5 [3–6.3]	** < 0.001**	–		
Mdz dose (mg/kg/day)	0.35 [0.1–0.7]	0.5 [0.2–0.6]	0.3 [0.1–0.7]	0.78	–		
Mdz duration (days)	2 [1–3]	2 [1.3–2.8]	2 [1–3]	0.96	–		
Dxd dose (mcg/kg/day)	7.5 [6.3–12.3]	7.1 [5.5–7.5]	25.4 [na]	0.5	–		
Dxd duration (days)	2 [1.8–2.5]	2 [2–3]	1 [na]	0.35	–		
ICU length stay (days)	3 [2–5]	3 [2–4]	4 [2.5–6]	0.063	–		
Duration of cEEG (h)	70.5 [25–141]	66 [22–96]	128 [93.5–169]	** < 0.001**	–		
Mechanical ventilation	64 (70%)	44 (64%)	20 (87%)	**0.036**	–		
Pa/Fio2	227 [162–184]	225 [158–283]	230 [167–301]	0.52	–		
Vasoactive drugs	76 (83%)	55 (80%)	21 (91%)	0.2	–		
Temperature (°C)	37.5 [37.1–38]	37.2 [36.6–37.9]	37.2 [37.1–37.6]	0.15	–		
Laboratory variables							
WBC	18.5 [13.3–26.8]	15 [10.4–23.2]	15.9 [12.3–24.6]	0.62	–		
Creatinine	1.5 [1.04–3.06]	1.4 [0.96–2.4]	1.76 [1.4–4]	0.15	–		
Bilirubin	1.05 [0.56–1.7]	0.9 [0.46–1.4]	0.73 [0.5–1.3]	0.16	–		
NSE	18.8 [13.9–30.5]	16 [13.5–25.3]	21.6 [13.9–30.4]	0.36	–		
Na^+^	137 [135–139]	137 [135–139]	136 [135–138]	0.46	–		
NH4^+^	78 [68–93]	71 [64–121]	85 [68–87]	0.93	–		
PaCO_2_	34 [30–38]	34 [30–37]	34 [32–38]	0.62	–		
Acute liver injury	26 (28%)	20 (29%)	6 (26%)	0.79	–		
Acute kidney injury	63 (68%)	47 (68%)	16 (70%)	0.89	–		
Chronic kidney injury	10 (11%)	5 (7%)	5 (22%)	0.053	–		
Antibiotics							
Beta-lactams	78 (85%)	59 (85%)	19 (83%)	0.74	–		
Cephalosporin	20 (22%)	14 (20%)	6 (26%)	0.56	–		
Metronidazole	14 (15%)	10 (14%)	4 (17%)	0.74	–		
Other	35 (38%)	24 (35%)	11 (45%)	0.26	–		

Data are presented as median [interquartile range] or count (percentage)

*APACHE-II* Acute physiological and chronic health evaluation, *CRS-R* The Coma Recovery Scale-Revised, *Pa/FiO2* Ratio of arterial oxygen partial pressure to fractional inspired oxygen, *CAM-ICU* The Confusion Assessment Method for the ICU, considered as positive if patients presented with delirium and RASS >  -4, *RASS* Richmond agitation sedation scale, *Mdz* Midazolam, *Dxd* Dexmedetomidine, *ICU* Intensive care unit, *(c)EEG* (Continuous) electroencephalogram, *T*° Body temperature, *WBC* White blood cells count, *NSE* Neuron-specific enolase, *NH4* Ammonemia in µg/dL, *PDs* Periodic discharges, *RDA* Rhythmic delta activity, *PDR* Posterior dominant rhythm. Mann–Whitney, Fisher’s exact and *χ*^2^ tests were used to analyze differences in variables between groups, as appropriate. *p* < 0.05 was considered statistically significant and marked in bold in the table. For the T° and the other laboratories variables (WBC, creatinine, bilirubin, NSE), the highest value during the ICU period was considered. For the definition of acute/chronic liver/kidney injury, please refer to the text

### EEG findings

**Fig. 1:**
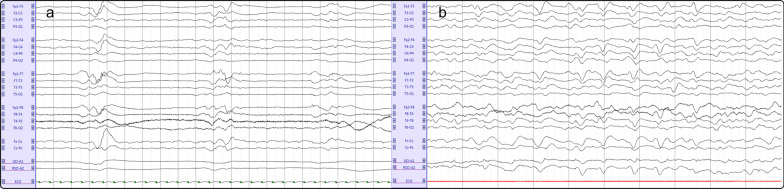
Representative examples of a suppression-burst EEG (panel **a**) and of an EEG showing generalized periodic discharges (panel **b**) in two septic ICU patients. Panel **a**: recording: 20 s, sensitivity: 50 μV/mm; bandpass filter frequencies 0.53–70 Hz, montage: longitudinal bipolar. Panel **b**: recording: 10 s, sensitivity: 70 μV/mm; bandpass filter frequencies 0.53–70 Hz, montage: longitudinal bipolar

cEEG was started within the first 24 h in 82 (89%) patients and within 48 h in 87 (95%)***;*** a total of 7431 h (309 days) of cEEG were visually analysed. Eighty-eight (96%) patients presented an abnormal background; 55 (59%) presented a low grade (I or II) and 33 (36%) a high grade (III or IV) encephalopathy. Burst-suppression (Fig. 1, panel a) and suppressed backgrounds were rare (2 [2%] and 1 [1%], respectively) and observed only in sedated patients. A low-voltage background was present in 9 patients (10%) and a discontinuous in 5 patients (5%). Twenty-two out of 26 (85%) patients with a normal clinical assessment showed some EEG background abnormalities, mainly theta slowing. We did not observe any electroencephalographic seizure.

Rhythmic delta activity was observed in 48 patients (52%), with a mean burden ranging from 1 to 30%. Periodic discharges (Fig. 1, panel b) were present in 23 patients (25%) with a mean burden ranging from 5 to 60%. Two patients presented lateralized PDs, and the remaining were generalized, all frontally predominant; the median PDs frequency was 1.4 [1–1.6] Hz. In univariate analysis (Table 1), the presence of PDs was associated with female gender (57 vs 25%, *p* = 0.005), a higher APACHE-II score (27 vs. 21, *p* = 0.049), a lower CRS-R score (5 vs. 22, *p* < 0.001) and GCS (7 vs 14, *p* < 0.001), the presence of SAE (96 vs. 64%, *p* = 0.003) and of sedation (57 vs. 25%, *p* = 0.006, particularly propofol regarding both total dose and duration, *p* < 0.001 for both). The same variables remained significantly associated with PDs considering only non-sedated patients (Additional file 1: Table S2). A trend was noted between PDs and higher NSE blood level (21.6 vs 16 ng/ml, *p* = 0.36). In multivariate analysis, female gender and the APACHE II were statistically associated with PDs (Table 1).

A cut-off of EEG-reactivity-percentage of 0.6 had the best accuracy to discriminate good functional outcome (specificity and sensitivity of 0.38 and 0.83, respectively). Using this cut-off, eighteen patients (19%) presented a mostly unreactive EEG. In univariate analysis, the absence of EEG reactivity was significantly associated with sedation, a lower CRS-R and GCS scores, a higher NSE value, the presence of PDs and the severity of the encephalopathy (Table 2). In multivariate analysis, sedation and the severity of encephalopathy were statistically associated with reactivity (*p* = 0.03 and *p* = 0.01 respectively). In non-sedated patients (Additional file 1: Table S3), only the severity of encephalopathy and the NSE level remained associated with reactivity.

**Table 2:** Clinical characteristics and EEG patterns associated with EEG reactivity

	Reactivity		*p* value
	Mostly present (*n* = 18)	Mostly present (*n* = 74)	
Age	69 [48.5–74]	65 [59–73]	0.95
Female	10 (56%)	20 (27%)	**0.021**
APACHE-II	23 [19–27]	21 [16–30]	0.33
non-neuro APACHE-II	14.5 [9–20]	19 [13–25]	0.053
CRS-R	1 [0–5]	22 [6–23]	** < 0.001**
GCS	3 [3–8]	14 [10–15]	** < 0.001**
CAM-ICU +	5/6 (83%)	24/64 (37.5%)	0.07
Sedation	14 (78%)	16 (22%)	** < 0.001**
T°	37.8 [37.4–38.6]	37.4 [37.5–37.9]	0.06
WBC (× 10^3^/mm^3^)	19.7 [14.5–32.6]	18.5 [12.5–25.6]	0.33
Creatinine (mg/dl)	1.58 [1.33–2.63]	1.5 [1.-3.09]	0.6
Bilirubin (mg/dl)	1.2 [0. 9–2]	0.9 [0.5–1.6]	0.12
NSE (ng/ml)	32.7 [21.2–41.8]	17.3 [13.6–27.1]	**0.012**
Acute kidney injury	13 (72%)	50 (67%)	0.7
Chronic kidney injury	3 (17%)	7 (9%)	0.4
Acute liver injury	6 (33%)	20 (27%)	0.59
EEG PDs	9 (50%)	14 (19%)	**0.006**
EEG RDA	13 (72%)	35 (47%)	0.06
High grade mSynek scale	15 (83%)	18 (24%)	** < 0.001**

Data are presented as median [interquartile range] or count (percentage)

*APACHE-II* Acute physiological and chronic health evaluation, *Non-neuro APACHE-II* APACHE II minus the GCS component, *CRS-R* The Coma Recovery Scale-Revised, *GCS* Glasgow coma scale, *CAM-ICU* The Confusion Assessment Method for the ICU, considered as positive if patients presented with delirium and RASS > -4, *T*° Body temperature, *WBC* White blood cells count, *NSE* Neuron-specific enolase, *PDs* Periodic discharges, *RDA* Rhythmic delta activity, *PDR* Posterior dominant rhythm, *High grade mSynek scale* High grade modified Synek scale defined as grade of III or more (please refer to Additional file 1: Table S1). Mann–Whitney, Fisher’s exact and *χ*^2^ tests were used to analyze differences in variables between groups, as appropriate. *p* < 0.05 was considered statistically significant and marked in bold in the table. For the T° and the other laboratories variables (WBC, creatinine, bilirubin, NSE), the highest value during the ICU period was considered

### Relationship of EEG findings with functional outcome

Thirty-seven (40%) patients presented an unfavourable functional outcome at hospital discharge. Parameters associated with functional outcome are summarized in Table 3. On univariate analysis, unfavourable outcome was associated with a higher APACHE II (26 vs. 20, *p* = 0.02), a lower CRS-R (3 vs. 22, *p* < 0.001) and GCS scores (7 vs 14, *p* < 0.001), and more frequent (84 vs. 64%, *p* = 0.035) and severe (high grade mSynek scale 60 vs. 20% *p* < 0.001) encephalopathy. On the contrary, neither the presence of a discontinuous/low voltage nor of a suppressed/suppression-burst background were associated with an unfavourable outcome (*p* = 0.09 and *p* = 0.06, respectively), albeit a tendency to prevail in that group of patients. Sedation, propofol infusion duration and mechanical ventilation were more frequent in patients with unfavourable outcome (49 vs. 22%, *p* = 0.007, 3 [2–4.5] vs 1 [1–2] days, *p* = 0.007 and 81 vs. 62%, *p* = 0.049, respectively). Unfavourable functional outcome was also associated with the occurrence of PDs (41 vs. 15%, *p* = 0.005) and PDs burden (range [0–60%] vs. [0–46%], *p* = 0.008). Furthermore, there was a negative correlation between the GOSE score and the PDs burden (Spearman’s coefficient *ρ* = − 0.2, *p* = 0.047). No significant correlation was identified between PDs frequency and GOSE scoring in patients with PDs (*ρ* = − 0.03, *p* = 0.88). Reactivity was prevalent in favourable functional outcome subjects (87 vs 70%, *p* = 0.04). In non-sedated patients (Additional file 1: Table S4), only APACHE II, CRS-R, GCS, high grade EEG encephalopathy, the presence of PDs and PDs burden remained statistically associated with outcome. The multivariate analysis confirmed that PDs were independently associated with an unfavourable outcome (OR 3.82, IC 95% [1.27–11.49]).

**Table 3:** Clinical, EEG and laboratory variables associated with functional outcome

	Functional outcome					
	Favourable (*n* = 55)	Unfavourable (*n* = 37)	Univariate	Multivariate		
			*p* value	*p* value	OR	CI 95%
Age (> 65y)	22 (40%)	22 (59%)	0.067	0.06	2.59	[0.96–7.02]
Female	18 (33%)	12 (32%)	0.97	–		
APACHE II	20 [15–24]	26 [21–33]	**0.02**	–		
non–neuro APACHE II	17 [10–23]	19 [14–25]	0.31	–		
APACHE II (> 23)	20 (36%)	21 (57%)	0.054	0.5	1.39	[053–3.66]
CAM-ICU +	18/47 (38%)	11/23 (49%)	0.45	–		
CRS-R	22 [11–23]	3 [1–18]	** < 0.001**	–		
GCS	14 [11–15]	7 [3–14]	** < 0.001**	–		
Sedation	12 (22%)	18 (49%)	**0.007**	–		
Propofol dose (mg/kg/day)	17 [3–47]	25 [15–35]	0.25	–		
Propofol duration (day)	1 [1–2]	3 [2–4.5]	**0.007**	–		
Mdz dose (mg/kg/day)	0.6 [0.6–1.5]	0.2 [0.1–04]	0.07	–		
Mdz duration (day)	2 [2–3.5]	1.5 [1–1.5]	0.27	–		
Dxd dose (µg/kg/day)	7.1 [na]	7.9 [6–16.7]	1	–		
Dxd duration (day)	2 [na]	2 [1–3]	1	–		
Vasoactive drugs	46 (84%)	30 (81%)	0.75	–		
Mechanical ventilation	34 (62%)	30 (81%)	**0.049**	–		
SAE	35 (64%)	31 (84%)	**0.035**	0.21	2.8	[0.66–6.52]
cEEG rhythmic and periodic patterns						
RDA	25 (45%)	23 (62%)	0.11	–		
PDs	8 (15%)	15 (41%)	**0.005**	**0.02**	3.82	[1.27–11.49]
PDs burden	[0–46%]	[0–60%]	**0.008**	–		
cEEG background						
Reactivity	48 (87%)	26 (70%)	**0.044**	–		
Sleep	36 (65%)	19 (51%)	0.17	–		
PDR	52 (95%)	30 (81%)	**0.042**	–		
High grade mSynek scale	11 (20%)	22 (60%)	**< 0.001**	–		
SB/suppressed	0 (0%)	3 (8%)	0.06	–		
Discontinuous/Low voltage	4 (7%)	7 (19%)	0.09	–		

Data are presented as median [interquartile range] or count (percentage)

*APACHE-II* Acute physiological and chronic health evaluation, *Non-neuro APACHE-II* APACHE II minus the GCS component, The APACHE-II > 23 represents the prevalence of APACHE scores above the median value of the study population (i.e., 22). *CAM-ICU* Confusion Assessment Method for the ICU, *CRS-R* The Coma Recovery Scale-Revised, *Mdz* Midazolam, *Dxd* Dexmedetomidine, *SAE* Sepsis-associated encephalopathy, *ICU* Intensive care unit, *(c)EEG* (continuous) electroencephalogram, *RDA* Rhythmic delta activity, *PD* Periodic discharges, *PDR* Posterior dominant rhythm, *High grade mSynek scale* High grade modified Synek scale defined as grade of III or more (please refer to Additional file 1: Table S1), *SB* Suppression-Burst, Discontinuous/Low Voltage/SB/suppressed background: please refer to text for definition. Mann–Whitney, Fisher's exact and *χ*^2^ tests were used to analyze differences in variables between groups, as appropriate. Multivariate logistic regression was performed. *p* < 0.05 was considered statistically significant and marked in bold in the table. After correction for disease severity, age and the presence of SAE, PDs occurrence was statistically associated with an unfavorable outcome (*p* = 0.02)

### Relationship between EEG abnormalities and mortality

Twenty-eight patients (33%) died during hospitalization. Parameters associated with mortality are summarized in Table 4. On univariate analysis, mortality was associated with age (64 vs. 41% of patients > 65 ys old, *p* = 0.037), APACHE II score (61 vs. 38% of patients with APACHE II score > 23, *p* = 0.039), CRS-R score (*p* = 0.001), GSC (*p* = 0.004) and the presence of SAE (89 vs. 23%, *p* = 0.013). Sedation tended to be more frequent in non-survivors, but the association was not statistically significant (46 vs. 23%, *p* = 0.06). Longer propofol infusion and lower median midazolam dose were associated with mortality (*p* = 0.009 and *p* = 0.03, respectively). Among EEG parameters, the presence of PDs and PDs burden was associated with a higher mortality (*p* = 0.009 and *p* = 0.014), in both sedated (Table 4) and non-sedated patients (Additional file 1: Table S5) and the same was noted for high grade EEG encephalopathy (61 vs 25%, *p* = 0.001). Reactivity was prevalent in survivors (*p* = 0.044) too, whereas neither the presence of a discontinuous/low voltage nor a suppressed/suppression-burst background was associated with mortality (*p* = 0.06 and *p* = 0.09, respectively), albeit a tendency to prevail in non-survivors. After correction for severity of brain and systemic dysfunction (i.e., APACHE II) in the multivariable analysis, the presence of PDs remained associated with mortality (OR 2.94, IC 95% [1.07–8.18], *p* = 0.038).

**Table 4:** Clinical, EEG and laboratory variables associated with in hospital mortality

	In hospital mortality					
	Non-survivors (*n* = 28)	Survivors (*n* = 64)	Univariate	Multivariate analysis		
			*p* value	*p* value	OR	CI 95%
Age (> 65y)	18 (64%)	26 (41%)	**0.037**	–		
Female	10 (36%)	20 (31%)	0.67	–		
APACHE II	27 [21–32]	21 [20–26]	**0.008**	0.059	1.05	[0.99–1.11]
non-neuro APACHE II	19 [15–24]	17 [10–23]	0.233	–		
APACHE II (> 23)	17 (61%)	24 (38%)	**0.039**	–		
CAM-ICU+	10/19 (53%)	19/51 (37%)	0.25	–		
CRS-R	3 [1–14]	22 [7–23]	**0.001**	–		
GCS	10 [5–15]	14 [8–15]	**0.004**	–		
Sedation	13 (46%)	17 (27%)	0.061	–		
Propofol dose (mg/kg/day)	24.2 [15–35.5]	18 [4–37]	0.36	–		
Propofol duration (day)	3 [2–6]	1 [1–3]	**0.009**	–		
Mdz dose (mg/kg/day)	0.1 [0.1–0.3]	0.6 [0.6–0.9]	**0.03**	–		
Mdz duration (day)	1.5 [1–2.75]	2 [2–3]	0.37	–		
Dxd dose (µg/kg/day)	3.4 [na]	7.9 [7.5–16.7]	0.5	–		
Dxd duration (day)	4 [na]	2 [1.5–2]	0.34	–		
Vasoactive drugs	24 (86%)	52 (81%)	0.6	–		
Mechanical ventilation	22 (79%)	42 (66%)	0.214	–		
SAE	25 (89%)	15 (23%)	**0.013**	–		
cEEG rhythmic and periodic patterns						
RDA	17 (61%)	31 (48%)	0.28	–		
PDs	12 (43%)	11 (17%)	**0.009**	**0.038**	2.94	[1.07–8.18]
PDs burden	[0–60]%	[0–30]%	**0.014**	–		
cEEG background						
Reactivity	19 (68%)	55 (86%)	**0.044**	–		
Sleep	13 (46%)	42 (66%)	0.08	–		
PDR	23 (82%)	59 (92%)	0.15	–		
High grade mSynek scale	17 (61%)	16 (25%)	**0.001**	–		
SB/suppressed	2 (7%)	1 (1%)	0.22	–		
Discontinuous/Low voltage	6 (21%)	5 (8%)	0.06	–		

Data are presented as median [interquartile range] or count (percentage)

*APACHE-II* Acute physiological and chronic health evaluation, *Non-neuro APACHE-II* APACHE II minus the GCS component, *CAM-ICU* Confusion Assessment Method for the ICU, *CRS-R* The Coma Recovery Scale-Revised, *Mdz* Midazolam, *Dxd* Dexmedetomidine, *SAE* Sepsis-associated encephalopathy, *ICU* Intensive care unit, *(c)EEG* (continuous) electroencephalogram, *RDA* Rhythmic delta activity, *PDs* Periodic discharges, *PDR* Posterior dominant rhythm, *High grade mSynek scale* High grade modified Synek scale defined as grade of III or more (please refer to Additional file 1: Table S1), *SB* Suppression-Burst; Discontinuous/Low voltage/Suppression-Burst/suppressed background: please refer to text for definition. Mann–Whitney, Fisher’s exact and *χ*^2^ tests were used to analyze differences in variables between groups, as appropriate. *p* < 0.05 was considered statistically significant and marked in bold in the table. Multivariate logistic regression was performed and showed that, after correction for disease severity, PDs occurrence negatively influenced survival

### Variables associated with SAE

Variables associated with SAE in both sedated (Additional file 1: Table S6) and non-sedated patients (Additional file 1: Table S7) were APACHE II score (*p* = 0.001 and *p* < 0.001, respectively), a longer duration of cEEG (*p* < 0.001 and *p* = 0.001, respectively), a higher prevalence of mechanical ventilation (*p* < 0.001 and *p* = 0.028, respectively), and the presence of PDs (*p* = 0.003 and *p* = 0.025, respectively).

## Discussion

In this cohort of highly selected septic patients without known brain injury or pre-existing encephalopathy admitted to an ICU, no seizures were observed on cEEG but a quarter of them presented PDs. The presence and the burden of PDs were independently associated with unfavourable functional outcome, including mortality, even after correction for disease severity.

 While the relationship between seizures and unfavourable functional outcome has already been established [11, 15–17], the question whether PDs negatively affect outcome in critically ill patients with a medical illness remains a matter of debate. Two prior studies yielded conflicting results [11, 17]. To the best of our knowledge, only one study specifically analysed this association in septic patients [12], showing that PDs are not a predictor of functional or cognitive outcome. However, in this study, PDs occurred in less severely ill patients, who received less sedation. Since disease severity is a predictor of functional outcome [11], we could speculate that the severity of the systemic organ dysfunction may have concealed the effect of PDs. Furthermore, authors suggest that sedatives drugs with anti-epileptic properties may have reduced PDs occurrence. In our cohort, the effect of PDs was independent from sedation and they remained a predictor of functional outcome even after correcting for possible confounders (i.e., age, APACHE II and the presence of encephalopathy). In support to this hypothesis, we also demonstrated that the PDs burden inversely correlated with the GOSE score, suggesting that not only the presence but also the duration of brain exposure to these patterns may play a role.

As for functional outcome, we showed that both PDs presence and PDs burden are associated with in-hospital death in both sedated and non-sedated patients. Epileptic abnormalities, such as seizures and PDs, are associated with mortality in critically ill patients [11], but this association is questioned in septic patients [12–14]. In particular, in two previous studies where continuous EEG was employed [12, 14], PDs were not associated with mortality. It has to be noted that, in our cohort, patient presenting with PDs had a more severe clinical condition and brain dysfunction. In fact, a tendency toward a higher NSE level was shown in patients with PDs. It is thus plausible that PDs partially reflect the severity of the underlying condition. However, the fact that, after correcting for disease severity, the PDs presence was still independently associated with mortality and SAE and that the burden of PDs also correlates with mortality, strengthens our hypothesis that there might be a causal correlation and that PDs may exert a negative effect on the brain. The pathophysiological changes that may explain the association between PDs and outcome are poorly understood. While seizure burden has already been robustly associated with functional outcome [15, 16], data about the effects of PDs burden on cerebral homeostasis are scarce, especially in non-neurological illness. A previous study in ICU patients showed that PDs lasting more than 24 h were associated with a worse outcome than transient PDs [17]. Since mechanisms of cerebral blood flow homeostasis (such as the neurovascular coupling [NVC] or pressure cerebral autoregulation) are altered in sepsis [26], our results may suggest that PDs may challenge an impaired system and cause brain hypoxia and potentially secondary brain injury. However, this hypothesis remains speculative since we did not record brain tissue metabolic or hemodynamic variables and sepsis induces many other brain alterations, from neuroinflammation and oxidative stress, to excitotoxicity and abnormal neuronal activation that might influence outcome together with hemodynamic alterations.

Of note, the association between mortality and lower doses of midazolam is probably due to the fact that those patients were already receiving other anaesthetics (namely propofol) that reduced the need for midazolam.

 In accordance with previous studies in septic patients [12–14], lack of EEG reactivity was associated with unfavourable outcome and mortality. Considering non-sedated patients, we showed that lack of reactivity probably reflects brain dysfunction since it was associated with higher grades of encephalopathy and brain damage (i.e., higher NSE, *p* = 0.04). The question whether lack of EEG reactivity per se might have a prognostic value, such as in cardiac arrest, warrants further studies.

 Prospective studies assessing EEG abnormalities in septic patients are scant. We confirmed, as previously reported [9], that cEEG is more sensitive than clinical criteria to detect encephalopathy since background abnormalities (essentially theta slowing) occur in at least 80% of patients with no clinical evidence of SAE. Although the prevalence of PDs was consistent with the literature (25% in [12], 22% in [13] and 19% in [14]), we did not record any seizures. This finding is not completely unexpected. In fact, while PDs are strongly associated with the occurrence of NCS [27] and sepsis has been considered as a risk factor for NCS [11], a history of prior neurological injury has also been associated with an increased risk of NCS [12]. The exclusion of patients with acute or remote neurological event (including cognitive or psychiatric disorders) likely contributed to the absence  of NCS, as also observed in another study that shared our including criteria [14]. These findings suggest that sepsis might be a seizure trigger in the presence of a pre-existing cerebral lesion. However, since our cohort presents a moderately severe sepsis, it may also be possible that seizures develop in more severe sepsis, cases that were less represented in our population. We also noticed an increased prevalence of female gender in the PDs group. This association has already been described in other brain injury settings, such as SAH [28], and ICU patients in general [11], but never reported in septic patients. Despite a known sex hormone influence on seizure occurrence [29], the reason for this finding is unclear.

As expected, SAE was prevalent in more severely affected patients, which were also more frequently sedated. The presence of RDA and, in part, the severity of the cEEG background abnormalities, were associated with SAE likely through the bias of sedation, as this association was absent in non-sedated patients. The only EEG abnormalities that remained associated with SAE occurrence even in non-sedated patients were PDs.

Our study presents strengths and limitations. This is the first prospective study systematically assessing the influence of PDs burden on outcome in a highly selected population of septic patients without prior or acute neurological disorder. Furthermore, inclusion criteria allowed to exclude most possible confounders in the definition of SAE. However, the small size of our single centre cohort may have introduced some selection bias. In particular, since our population included septic patients with a moderately severe infection, our results may not be immediately transferable to a more severely affected population. Moreover, our definition of SAE is unusual. There is no specific clinical assessment tool to diagnose SAE. The CAM-ICU, a frequently employed scale, has been developed to diagnose delirium [30], but SAE may present with a broader clinical spectrum than just delirium [31]. In addition, the CAM-ICU is not assessable if RASS < − 3, even if comatose non-sedated patients evidently present with a brain dysfunction. Thus, we looked for a scale which might identify subtle alteration in cognition, such as attention deficit or fluctuation in vigilance, which CAM-ICU may underestimate. CRS-R might best capture these features, as it integrates the variability in patients’ responses throughout the assessment, which may take up to 30 min [22]. Furthermore, since we included also sedated patients, drugs might have influenced the prevalence of EEG abnormalities. Nevertheless, since the association between PDs, PDs burden, functional outcome and mortality remained statistically significant in non-sedated patients too, sedative drugs did not probably influence the main findings of our work. We did not systematically perform cerebral imaging since patients did not present with focal neurological signs, which are more frequently associated with ischemic lesions and correlate with mortality [32]. However, it would have been interesting to look for a correlation between PDs presence and acute brain lesions (i.e., ischemic), which might have furtherly supported the deleterious effect of PDs. Further studies should also analyse the long-term cognitive outcome of septic patients presenting with PDs.

## Conclusions

In this prospective study, we found that the presence and burden of PDs were independently associated with an unfavourable functional outcome and mortality in septic patients. Furthermore, PDs were independently associated with SAE, suggesting that they might contribute to the brain dysfunction observed in septic patients. These data suggest an important role for cEEG to detect these abnormalities in septic encephalopathic patients.

## Supplementary Information

**Additional file 1.** It contains additional tables.

## Data Availability

The data underlying this article will be shared on reasonable request to the corresponding author.

## Abbreviations

APACHE II: Acute Physiologic and Chronic Health Evaluation
CAM-ICU: Confusion Assessment Method for the ICU
CRS-R: Coma Recovery Scale-Revised
(c)EEG: (Continuous) electroencephalogram
GOSE: Glasgow Outcome Scale-Extended
NCS: Nonconvulsive seizure
NSE: Neuron-specific enolase
PDs: Periodic discharges
SAE: Sepsis-associated encephalopathy
