# Supplementary material for: The association between the presence and burden of periodic discharges and outcome in septic patients: an observational prospective study
Source: Crit Care. 2023 May 9;27:179. doi: 10.1186/s13054-023-04475-w (PMC10170680; doi:10.1186/s13054-023-04475-w)
Supplement: Supplementary file 1 — Additional file 1. It contains additional tables. [file 13054_2023_4475_MOESM1_ESM.docx]

**Table S1. Modified Synek scale for encephalopathy grading based on EEG background.**

|  | **Grade 0** | **Grade I** | **Grade II** | **Grade IIIa** | **Grade IIIb** | **Grade IV** | **Grade V** |
| --- | --- | --- | --- | --- | --- | --- | --- |
| **Synek** | **NA** | **Grade I** | **Grade II** | **Grade IIIa** | **Grade IIId** | **Grade IIb, IIIb-c, IV** | **Grade V** |
| **Predominant background frequency** | α | α | θ, α | δ, θ | δ | δ or rhythmic (α, spindle, b, θ) coma pattern or SB or GPDs-suppression | NA |
| **Additional activities** | β, occasional θ | θ | α, δ | θ, α | θ | variable | NA |
| **PDR** | ≥8.5 | ≥8.5 | <8.5 | absent | absent | absent | NA |
| **Amplitude** | N (or β only) | N | N | N | N or Attenuation | Attenuation | Suppression |
| **Continuity** | C | C | C | C/NC | C/NC>DC | NC/DC/BS | NA |
| **Variability** | V | V | V | V | Minimally variable | NV | NA |
| **Reactivity** | R | R/SIRPIDs | R/SIRPIDs | R/SIRPIDs | Minimally R/SIRPIDs | NR/SIRPIDs | NA |

Abbreviations: δ: EEG oscillation frequency < 4Hz, θ: 4-8, α: 8-12 Hz, β: 12 Hz, N: normal, NA: not assessable, PDR: posterior dominant rhythm, C: continuous, NC: nearly continuous, DC: discontinuous, BS: bursts suppression (according to ^1^) , V: variable, NV: non variable, R: reactive, SIRPIDs: stimulus induced rhythmic, periodic, or ictal discharges. The presence of a PDR defines the low-grade encephalopathies (grade I/II). ***Reactivity*** was tested once daily following a standardized protocol: calling the patient’s name quietly, then loudly, followed by gentle tactile stimulation, followed by noxious stimulation (nail bed pressure) and finally passive eyes opening. The presence of a PDR, attenuating with eyes opening, also defined the EEG background as reactive.

**Table S2. Clinical and laboratory parameters associated with periodic discharges in non-sedated patients.**

| **Parameters** | **Periodic discharges without sedation** | | | | **p value** |
| --- | --- | --- | --- | --- | --- |
|  | **Absent** (n=52) median [IQR]  n (%) | | **Present** (n=10) median [IQR]  n (%) | |  |
| Age | 66.5 | [60-74] | 72.5 | [60.5-75.5] | 0.56 |
| Female | 14 | (27%) | 7 | 70%) | **0.024** |
| APACHE II | 20 | [19-23] | 30 | [27-32] | **0.009** |
| APACHE II > 23 | 22 | (42%) | 8 | (80%) | **0.04** |
| CRS-R | 22 | [19-23] | 7 | [2-16] | **0.001** |
| SAE | 27 | (52%) | 9 | (90%) | **0.035** |
| GCS | 15 | [14-15] | 11 | [8-14] | **0.007** |
| CAM-ICU + | 15/50 | (30%) | 7/9 | (78%) | **0.006** |
| RASS < -3 | 2 | (4%) | 3 | (30%) | 0.42 |
| T | 37.4 | [37-38] | 37.5 | [37.2-37.8] | 0.37 |
| WBC | 15.9 | [12.2-23.5] | 18.3 | [13.1-37.2] | 0.5 |
| Creatinine | 1.4 | [0.9-2.55] | 2.65 | [1.52-4.38] | 0.051 |
| Bilirubin | 0.89 | [0.37-1.5] | 1.65 | [0.66-2.08] | 0.059 |
| NSE | 16 | [15.5-32.5] | NA | NA | NA |
| Acute kidney injury | 34 | (65%) | 7 | (70%) | 1 |
| Chronic kidney injury | 4 | (8%) | 2 | (20%) | 0.24 |
| Acute liver injury | 12 | (23%) | 5 | (50%) | 0.12 |

Data are presented as median [IQR] or n (%). Abbreviations: APACHE-II: Acute Physiological and Chronic Health Evaluation; Non-neuro APACHE-II: APACHE II minus the GCS component; CRS-R: the Coma Recovery Scale-Revised; GCS: Glasgow Coma Scale; CAM-ICU: The Confusion Assessment Method for the ICU, considered as positive if patients presented with delirium and RASS < -4; RASS: Richmond Agitation Sedation Scale; T°: body temperature; WBC: white blood cells count; NSE: neuron specific enolase; PDs: periodic discharges; RDA: rhythmic delta activity; PDR: posterior dominant rhythm. Mann Whitney, Fisher's exact and χ2 tests were used to analyze differences in variables between groups, as appropriate. P<0.05 was considered statistically significant and marked in bold in the table. For the T° and the other laboratories variables (WBC, creatinine, bilirubine, NSE), the highest value during the ICU period was considered.

**Table S3. Clinical characteristics and EEG patterns associated with EEG reactivity in non-sedated patients.**

|  | **Reactivity** | | | | **P value** |
| --- | --- | --- | --- | --- | --- |
|  | **Absent** (n=4) | | **Present** (n=58) | |  |
| Age | 81 | [71-84] | 66 | [60-74] | 0.12 |
| Female | 2 | (50%) | 19 | (3%) | 0.59 |
| APACHE-II | 20 | [14-29] | 22 | [16-30] | 0.72 |
| non-neuro APACHE-II | 13 | [8-21] | 19 | [15-26] | 0.31 |
| CRS-R | 6 | [4-11] | 22 | [18-23] | 0.16 |
| GCS | 8 | [7-10] | 14 | [13-15] | 0.13 |
| CAM-ICU + | 3/4 | (75%) | 33/57 | (58%) | 0.63 |
| T° | 37.3 | [37.3-37.6] | 37.4 | [37-38] | 0.98 |
| WBC (*10^3^/mm^3^) | 20 | [10.6-30.8] | 16 | [12.4-23] | 0.92 |
| Creatinine (mg/dl) | 3 | [1.7-4.3] | 1.5 | [0.9-3] | 0.17 |
| Bilirubine (mg/dl) | 8 | [1-22] | 0.9 | [0.4-1.6] | 0.08 |
| NSE (ng/ml) | 31.2 | [27.8-50] | 15.4 | [12.8-25.4] | **0.04** |
| EEG PDs | 2 | (50%) | 8 | (14%) | *0.05* |
| EEG RDA | 4 | (100%) | 23 | (40%) | **0.031** |
| High grade mSynek scale | 3 | (75%) | 7 | (12%) | **0.012** |

Data are presented as median [interquartile range] or count (percentage). Abbreviations: APACHE-II: Acute Physiological and Chronic Health Evaluation; Non-neuro APACHE-II: APACHE II minus the GCS component; CRS-R: the Coma Recovery Scale-Revised; GCS: Glasgow Coma Scale; CAM-ICU: The Confusion Assessment Method for the ICU, considered as positive if patients presented with delirium and RASS < -4; T°: body temperature; WBC: white blood cells count; NSE: neuron specific enolase; PDs: generalized periodic discharges; RDA: generalized rhythmic delta activity; PDR: posterior dominant rhythm; High grade mSynek scale : high grade modified Synek scale defined as grade of III or more (please refer to table S1, additional file). Mann Whitney, Fisher's exact and χ2 tests were used to analyze differences in variables between groups, as appropriate. P<0.05 was considered statistically significant and marked in bold in the table. For the T° and the other laboratories variables (WBC, creatinine, bilirubin, NSE), the highest value during the ICU period was considered.

**Table S4. Clinical and laboratory parameters associated with functional outcome in non-sedated patients.**

| **Parameters** | | **functional outcome** | | | | |
| --- | --- | --- | --- | --- | --- | --- |
|  |  | Favorable  (n = 43) | | Unfavorable (n = 19) | | **univariate** |
|  |  | median [IQR]/ n (%) | | median [IQR]/ n (%) | | p value |
| Age (> 65y) |  | 21 | (49%) | 12 | (63%) | 0.29 |
| Female |  | 14 | (33%) | 7 | (37%) | 0.74 |
| APACHE |  | 20 | [15-25] | 29 | [20-33] | **0.014** |
| non-neuro APACHE II | | 19 | [14-24] | 22 | [17-31] | 0.13 |
| APACHE II (> 23) | | 18 | (42%) | 12 | (63%) | 0.12 |
| GCS |  | 15 | [14-15] | 14 | [7-15] | **0.026** |
| CAM-ICU + |  | 15/42 | (36%) | 7/17 | (41%) | 0.69 |
| CRS-R |  | 22 | [19-23] | 18 | [3-23] | **0.03** |
| Vasoactive drugs | | 34 | (79%) | 12 | (63%) | 0.19 |
| Mechanical ventilation | | 22 | (51%) | 12 | (63%) | 0.38 |
| SAE |  | 23 | (53%) | 13 | (68%) | 0.27 |
| cEEG | RDA | 16 | (37%) | 11 | (58%) | 0.13 |
|  | PDs | 4 | (9%) | 6 | (32%) | **0.028** |
|  | PDs burden [range] | [0- | 20]% | [0- | 30]% | **0.036** |
|  | Reactivity | 41 | (95%) | 17 | (89%) | 0.58 |
|  | Sleep II | 27 | (63%) | 10 | (53%) | 0.45 |
|  | PDR | 43 | (100%) | 18 | (95%) | 0.13 |
|  | High grade mSynek scale | 3 | (7%) | 7 | (37%) | **0.003** |

Data are presented as median [IQR] or n (%). Abbreviations: APACHE-II: Acute Physiological and Chronic Health Evaluation; Non-neuro APACHE-II: APACHE II minus the GCS component; CAM-ICU: Confusion Assessment Method for the ICU; CRS-R: the Coma Recovery Scale-Revised; SAE: sepsis associated encephalopathy; ICU: Intensive Care Unit; (c)EEG: (continuous) electroencephalogram; RDA: rhythmic delta activity; PDs: periodic discharges; PDR: posterior dominant rhythm; High grade mSynek scale : high grade modified Synek scale defined as grade of III or more (please refer to table S1, additional file). The APACHE-II > 23 represents the prevalence of APACHE scores above the median value of the study population (i.e. 22). Data are presented as median [interquartile range or range] or count (percentage). Mann Whitney, Fisher's exact and χ2 tests were used to analyze differences in variables between groups, as appropriate. P<0.05 was considered statistically significant and marked in bold in the table.

**Table S5. Clinical and laboratory parameters associated with in hospital mortality in non-sedated patients.**

| **Parameters** | | **In-hospital death** | | | | |
| --- | --- | --- | --- | --- | --- | --- |
|  |  | non-survivors (n = 15) | | survivors (n = 47) | | **univariate** |
|  |  |  | |  | | p-value |
| Age (> 65y) |  | 10 | (67%) | 23 | (49%) | 0.23 |
| Female |  | 7 | (47%) | 14 | (30%) | 0.23 |
| APACHE II |  | 29 | [20-32] | 21 | [16-27] | *0.051* |
| non-neuro APACHE II | | 22 | [17-29] | 19 | [14-24] | 0.28 |
| GCS |  | 13 | [7-14] | 15 | [14-15] | **0.006** |
| CAM-ICU + |  | 7/14 | (50%) | 15/45 | (30%) | 0.26 |
| CRS-R |  | 6 | [3-22] | 22 | [19-23] | **0.006** |
| Vasoactive drugs | | 11 | (73%) | 35 | (74%) | 0.93 |
| Mechanical ventilation | | 9 | (60%) | 25 | (53%) | 0.65 |
| SAE |  | 12 | (80%) | 24 | (51%) | 0.071 |
| EEG | RDA | 9 | (60%) | 18 | (38%) | 0.14 |
|  | PDs | 6 | (40%) | 4 | (9%) | **0.009** |
|  | PDs burden | [0- | 20]% | [0- | 30]% | **0.006** |
|  | Reactivity | 13 | (87%) | 45 | (96%) | 0.24 |
|  | Sleep | 7 | (47%) | 30 | (64%) | 0.24 |
|  | PDR | 14 | (93%) | 47 | (100%) | 0.074 |
|  | High grade mSynek scale | 6 | (40%) | 4 | (8%) | **0.004** |

Data are presented as median [IQR] or n (%). Abbreviations: APACHE-II: Acute Physiological and Chronic Health Evaluation; Non-neuro APACHE-II: APACHE II minus the GCS component; CAM-ICU: Confusion Assessment Method for the ICU; CRS-R: the Coma Recovery Scale-Revised; SAE: sepsis associated encephalopathy; ICU: Intensive Care Unit; (c)EEG: (continuous) electroencephalogram; RDA: rhythmic delta activity; PDs: periodic discharges; PDR: posterior dominant rhythm; High grade mSynek scale : high grade modified Synek scale defined as grade of III or more (please refer to table S1, additional file). The APACHE-II > 23 represents the prevalence of APACHE scores above the median value of the study population (i.e. 22). Data are presented as median [interquartile range or range] or count (percentage). Mann Whitney, Fisher's exact and χ2 tests were used to analyze differences in variables between groups, as appropriate. P<0.05 was considered statistically significant and marked in bold in the table.

**Table S6. Clinical and laboratory parameters associated with sepsis-associated encephalopathy.**

| Parameters | | **Sepsis-associated encephalopathy** | | | | **p** | **multivariate analysis** | | |
| --- | --- | --- | --- | --- | --- | --- | --- | --- | --- |
|  |  | **absent** (n=26) | | **present** (n=66) | |  | p | OR | IC 95% |
| Age (years) |  | 67 | [61.3-74] | 65 | [52.5-72.8] | 0.28 | - |  |  |
| Female |  | 5 | (19%) | 25 | (38%) | 0.15 | - |  |  |
| Sepsis origine |  |  |  |  |  | 0.79 | - |  |  |
|  | abdominal | 9 | (35%) | 28 | (42%) |  | - |  |  |
|  | respiratory | 7 | (27%) | 20 | (30%) |  | - |  |  |
|  | urinary | 4 | (15%) | 6 | (9%) |  | - |  |  |
|  | soft tissue | 4 | (15%) | 5 | (8%) |  | - |  |  |
|  | unknown | 1 | (4%) | 4 | (6%) |  | - |  |  |
|  | other | 1 | (4%) | 1 | (2%) |  | - |  |  |
| APACHE II |  | 17 | [13-24] | 24 | [19-30] | **0.001** | **0.019** | 1.08 | [1.02-1.15] |
| non-neuro APACHE | | 17 | [13-24] | 18 | [11-24] | 0.81 | - |  |  |
| APACHE II > 23 | | 7 | (27%) | 34 | (52%) | **0.033** |  |  |  |
| Days to EEG (range) | | 0 | [0-2] | 0 | [0-2] | 0.87 | - |  |  |
| Duration of cEEG (h) | | 22 | [4.5-47] | 93.5 | [45.3-163] | **< 0.001** | - |  |  |
| Sedation |  | 0 | (0%) | 30 | (45%) | **< 0.001** | - |  |  |
| Mechanical ventilation | | 10 | (38%) | 54 | (82%) | **< 0.001** | - |  |  |
| Vasoactive drugs | | 17 | (65%) | 59 | (89%) | **0.006** | - |  |  |
| Beta-lactams | | 22 | (85%) | 56 | (85%) | 0.9 |  |  |  |
| Cephalosporin | | 3 | (11%) | 17 | (26%) | 0.14 |  |  |  |
| Metronidazole | | 3 | (11%) | 11 | (17%) | 0.5 |  |  |  |
| Other | | 6 | (23%) | 29 | (44%) | 0.06 |  |  |  |
| Pa/Fio2 |  | 271 | [205-299] | 195 | [150-278] | **0.015** | - |  |  |
| Creatinine at admission | | 1.3 | [0,85-2,35] | 1.5 | [1-2,3] | 0.56 | - |  |  |
| ICU length of stay (days) | | 3 | [2-5] | 3 | [1-5] | 0.62 | - |  |  |
| Mortality hospitalisation | | 3 | (12%) | 25 | (38%) | **0.013** | - |  |  |
| Good functional outcome | | 20 | (77%) | 34 | (52%) | **0.031** | - |  |  |
| EEG | RDA | 9 | (35%) | 39 | (59%) | **0.034** | - |  |  |
|  | PDs | 1 | (4%) | 22 | (33%) | **0.003** | **0.04** | 8.98 | [1.11-72.8] |
|  | Reactivity | 25 | (96%) | 49 | (74%) | **0.017** | - |  |  |
|  | Sleep | 14 | (54%) | 41 | (62%) | 0.46 | - |  |  |
|  | PDR | 26 | (100%) | 53 | (80%) | **0.036** | - |  |  |

Data are presented as median [IQR] or n (%). Abbreviations: APACHE-II: Acute Physiological and Chronic Health Evaluation; Non-neuro APACHE-II: APACHE II minus the GCS component; ICU: intensive care units; PDs: generalized periodic discharges; GRDA: generalized rhythmic delta activity; PDR: posterior dominant rhythm. Mann Whitney, Fisher's exact and χ2 tests were used to analyze differences in variables between groups, as appropriate. P<0.05 was considered statistically significant and marked in bold in the table.

**Table S7. Clinical and laboratory parameters associated with sepsis-associated encephalopathy in non-sedated patients.**

| **Parameters** | | **Sepsis-associated encephalopathy** | | | | | **p value** | |
| --- | --- | --- | --- | --- | --- | --- | --- | --- |
|  |  | **Absent** (n=26) median [IQR]/ n (%) | | **Present** (n=36) median [IQR]/ n (%) | |  | |  |
| Age (years) |  | 67 | [61.3-74] | 67 | [54-75] | 0.28 | |  |
| Female |  | 5 | (19%) | 16 | (44%) | **0.038** | |  |
| Sepsis origin |  |  |  |  |  | 0.97 | |  |
|  | abdominal | 9 | (35%) | 16 | (44%) |  |  |  |
|  | respiratory | 7 | (27%) | 8 | (22%) |  |  |  |
|  | urinary | 4 | (15%) | 5 | (14%) |  |  |  |
|  | soft tissue | 4 | (15%) | 4 | (11%) |  |  |  |
|  | unknown | 1 | (4%) | 2 | (6%) |  |  |  |
|  | other | 1 | (4%) | 1 | (3%) |  |  |  |
| APACHE II |  | 17 | [13-24] | 26 | [20-30] | **< 0.001** | |  |
| non-neuro APACHE | | 17 | [13-24] | 22 | [16-29] | 0.07 | |  |
| Days to EEG (range) | | 0 | [0-2] | 0 | [0-2] | 0.4 | |  |
| Duration of cEEG (h) | | 22 | [4.5-47] | 69.5 | [35.5-102] | **0.001** | |  |
| Mechanical ventilation | | 10 | (38%) | 24 | (67%) | **0.028** | |  |
| Vasoactive drugs | | 17 | (65%) | 29 | (81%) | 0.18 | |  |
| Pa/Fio2 |  | 271 | [205-299] | 223 | [164-300] | 0.17 | |  |
| Creatinine at admission | | 1.3 | [0.85-2.35] | 1.5 | [01.07-3.35] | 0.42 | |  |
| ICU length of stay (days) | | 3 | [2-5] | 2 | [1-3] | 0.62 | |  |
| Mortality hospitalization | | 3 | (12%) | 12 | (33%) | **0.048** | |  |
| Good functional outcome | | 20 | (77%) | 23 | (64%) | 0.27 | |  |
| EEG | RDA | 9 | (35%) | 18 | (50%) | 0.23 | |  |
|  | PDs | 1 | (4%) | 9 | (25%) | **0.025** | |  |
|  | Reactivity | 25 | (96%) | 33 | (92%) | 0.63 | |  |
|  | Sleep | 14 | (54%) | 23 | (64%) | 0.43 | |  |
|  | PDR | 26 | (100%) | 35 | (97%) | 0.39 | |  |

Data are presented as median [IQR] or n (%). Abbreviations: APACHE-II: Acute Physiological and Chronic Health Evaluation; Non-neuro APACHE-II: APACHE II minus the GCS component; ICU: intensive care units; PDs: generalized periodic discharges; GRDA: generalized rhythmic delta activity; PDR: posterior dominant rhythm. Mann Whitney, Fisher's exact and χ2 tests were used to analyze differences in variables between groups, as appropriate. P<0.05 was considered statistically significant and marked in bold in the table.
